# Studying the Anticancer Effects of Thymoquinone on Breast Cancer Cells through Natural Killer Cell Activity

**DOI:** 10.1155/2022/9218640

**Published:** 2022-09-20

**Authors:** Huda F. Alshaibi, Nouf A. Aldarmahi, Nuha A. Alkhattabi, Hadeil M. Alsufiani, Nesrin I. Tarbiah

**Affiliations:** ^1^Biochemistry Department, Faculty of Sciences, King Abdulaziz University, Jeddah, Saudi Arabia; ^2^Embryonic Stem Cell Unit, King Fahd Medical Research Center, King Abdulaziz University, Jeddah, Saudi Arabia; ^3^Experimental Biochemistry Unit, King Fahd Medical Research Center, King Abdulaziz University, Jeddah, Saudi Arabia

## Abstract

Cancer immunotherapy is quickly growing and can now be viewed as the “fifth column” of cancer treatment. In addition, cancer immunotherapy has shown promising results with different kinds of cancers and may be used as a complementary therapy with various types of treatments. Thus, “immuno-oncology” is showing astounding advantages. However, one of the main challenges that face this type of therapy is that cancer cells can evade immune system elimination through different mechanisms. Many studies were done to overcome this issue including adding immune stimulants to generate synergistic effects or by genetically modifying NK cells themselves to be stronger and more resistant. *Nigella sativa*, also known as black cumin, is a well-known example of a widely applicable herbal medicine. It can effectively treat a variety of diseases, such as hypertension, diabetes, bronchitis, gastrointestinal upset, and cancer. The anticancer qualities of *Nigella sativa* appear to be mediated by an immune-modulatory effect that stimulates human natural killer (NK) cells. These are a type of lymphocyte and first line of defense against pathogens. *Objectives*. In this study, we investigated the therapeutic effect of thymoquinone, a major component of *Nigella sativa*, on the cytotoxic pathways of NK cells. *Methods*. NK cells were cultured with breast cancer cell line Michigan Cancer Foundation-7 (MCF-7); and were treated with Thymoquinone. The cytotoxicity of NK cells on cancer cells was measured. The cultured media were then collected and measured via enzyme-linked immunosorbent assay (ELISA) for concentrations of perforin, granzyme B and interferon-*α* (IFN-*α*). *Results*. The cytotoxic effect of NK cells on tumor cells was increased in the presence of thymoquinone, with an increased release of perforin, granzyme B, and IFN-*α*. *Conclusion*. Thymoquinone promotes the cytotoxic activity of NK cells against breast cancer MCF-7 cells.

## 1. INTRODUCTION

Breast cancer is one of the main causes of death in women according to the world health organization [1]. It is a metastatic and commonly spreads from its origin to distant organs of the body, with the most common sites of distant metastases being bone, liver, lungs, and brain [2]. Breast cancer is a heterogeneous disease [3] at the histological and biological levels due to genetic, epigenetic, and transcriptome changes. This phenotypic difference influences breast cancer diagnosis, treatment, and thus prognosis. Thus, initially treatment of breast cancer was depending on tumor characteristics such as its clinical stage, histopathologic features, and biomarker profiling. However, in the last few decades, our understanding of its biological and molecular characteristics has improved [4]. We can now classify breast cancer to five subtypes according to molecular profiling, hormone indicators, and growth factor expressions. These subtypes are luminal A and B, human epidermal growth factor receptor 2 (HER2) enriched, triple-negative or basal-like (BL), and normal- like BC. Luminal A subtype is characterized by high expression of luminal gene and hormone receptors genes including estrogen receptor (ER) and progesterone receptors (PR). Luminal B subtype is characterized by expressing luminal gene and moderate to low expression of both ER/PR genes. HER2 subgroup is characterized by high expression of HER2 and low expression of ER and related genes. Triple negative or BL are characterized by high expression of basal epithelial genes and basal cytokeratins, low expression of ER and related genes, and low expression of HER2 [4, 5] . The luminal A subtype presents a greater prognosis and higher survival rate than the luminal B subtype [6–8] . While on the other hand, it has been indicated that the HER2 subtype, characterised by positivity for HER2, is linked with aggressive histological characters, poor prognosis, and unresponsiveness to normal treatments and decreased in survival rate [9, 10]. This outcome may change dramatically when chemotherapy is used in combination with anti-HER2 monoclonal antibodies and tyrosine kinase inhibitor [11, 12]. Thus ER, PR, and HER2 are well established breast cancer biomarkers that aid in the diagnoses and treatment prognosis [4, 5]. Recently, some studies suggested the involvement of matrix metalloproteinase 2 (MMP-2) and matrix metalloproteinase 9 (MMP-9), also known as gelatinase A and gelatinase B, respectively, in breast cancer initiation and growth throughout complex interactions with the key oncogenes and tumor-suppressor genes which are involved in the initial stage of tumorigenesis. Consequently, several authors have proposed MMP-2 and MMP-9 as promising prognostic markers that may be used in early detection and treatment [13, 14].

Cancer immunotherapy [15] is quickly progressing and can now be viewed as the “fifth column” of cancer treatment, joining medical surgery, cytotoxic chemotherapy, radiation, and targeted treatment [16]. In addition, Cancer Immunotherapy (CI) has shown promising results with different kinds of malignancies and may be used in combination with various treatments. Thus, “immuno-oncology” is showing astounding advantages [17, 18].

NK cells are a type of lymphocyte that plays a central role in the innate immune response against pathogens and tumors [19–21]. These cells can rapidly identify and attack infected and malignant cells [22, 23]. CD56 and CD16 cells constitute around 90% of the circulating NK cell population, inducing cell lyses via cytolytic granules containing perforin and granzyme. These work synergistically with interferon-*α* (IFN-*α*) to induce apoptosis in target cells [24, 25]. NK cells have also been found to enhance antibody-mediated cytotoxicity, where NK triggers the release of specific antibodies in the immune system that recognize certain antigens in the invading pathogens [26]. NK cells instantly target pathogens or foreign materials via different mechanisms, one of the most important mechanisms is the perforin/granzyme apoptotic pathway, and other mechanism is tumor necrosis factor, as mediated by the interaction between the FAS ligands on NK cells with the death receptors (FAS/CD95) on target cells [27].

According to the literature, NK cell–based immunotherapy is a promising treatment strategy that can be used in adjuvant chemotherapy for both solid and hematologic tumors. This treatment protocol could combine surgery, chemotherapy, radiation, and monoclonal antibodies (mAb) [28]. However, many studies showed that NK cell activity decreases during the progression of many tumors including breast cancer especially those with functional estrogen receptors. Estrogen suppress NK cell activity by inhibiting NK cells activating receptors such as CD69, NKp46, NKG2D, and CD244, thus inhibiting NK cell activation and reducing the secretion of granzyme B, as well as FasL [29, 30].

*Nigella sativa*, also known as black cumin, it is a dicotyledon of the Ranunculaceae family, and has been used for two millennia as an appetizer, flavoring agent, and nourishing and nutraceutical substance in various societies in Asia, Africa, and Europe. Interestingly, *Nigella sativa* is a well-known example of a widely applicable herbal medicine [31]. Thymoquinone is a major active component of *Nigella sativa* [32]. As a heavenly panacea, *Nigella sativa* L. (Ranunculaceae) has attracted attention in traditional medication, as well as in present-day therapeutic exploration [33]. Research found that *Nigella sativa has* anticancer effects through mediated an immune-modulatory effect, which stimulates human natural killer (NK) cells [34].

Further investigations are still needed to understand the role of *Nigella sativa* in adjuvant chemotherapy at the molecular level [28]. However, its role in treating malignant cancer specifically is quite complicated, which can be ascribed to its multiple suppressive effects on proliferative cells, free radicals, mutagens and metastasis [15]. In this study, we examined the effect of thymoquinone, a component making up 30% of *Nigella sativa* [35], on cytotoxic pathways and the behavior and activity of NK cells. This may help in understanding the possible therapeutic role of thymoquinone on NK cell activity.

## 2. Methods

### 2.1. NK Isolation from PBMCs

Blood samples were collected from healthy volunteers in ethylene diamine tetra-acetic acid (EDTA) tubes (Xinlehj, India). The study was approved by the ethical committee of the Faculty of Medicine, King Abdulaziz University (ref. no. 640-20). Peripheral blood mononuclear cells (PBMCs) from healthy individuals are isolated by Ficoll density gradient centrifugation of peripheral venous blood (Sigma-Aldrich). NK cells are purified by positive selection of CD56 cells (CD56 MicroBeads, human, 130-050-401, Miltenyi biotec) and MACS column (130-042-201, Miltenyi biotec, USA) was used. In brief, the magnetically labelled CD56 + are retained within the column, the unlabeled cells run through; this cell fraction is thus depleted of CD56+ cells. After removing the column from the magnetic field, the magnetically retained CD56 + cells can be eluted as the positive selected cell. The purity of NK cells was 95.9% as assessed by flow cytometric analysis of cells stained with CD56-PE (DAKO, USA) (Novocyte Flow Cytometer; King Faisal Specialist Hospital & Research Centre, Jeddah, Saudi Arabia) Figure S1.

### 2.2. Thymoquinone Preparation

Thymoquinone (274666-1G, Sigma-Aldrich, USA) was prepared by dissolving 0.4926% thymoquinone crystal in 1% dimethyl sulfoxide anhydrous, ≥99.9% (276855, Sigma -Aldrich, USA) and 99.9% NK media (130-092-657, Miltenyi biotec, USA). Two dilutions of the extract, 25 and 50 *μ*M, were prepared and stored at 2–8°C.

### 2.3. Cell Lines and Tissue Culture

Human breast cancer MCF-7 (Michigan Cancer Foundation-7; King Faisal Specialist Hospital & Research Centre, Jeddah, Saudi Arabia) is an epithelial invasive breast ductal carcinoma cell line that is oestrogen–and progesterone receptor–positive. MCF-7 cells were cultured in RPMI1640 (2242279, Gibco, USA) and supplemented with 10% penicillin-streptomycin (10,000 U/mL; 15140122, Sigma-Aldrich, USA). The NK cells were cultured in an NK MACS medium (Miltenyi Biotec, USA) and supplemented with 20% Fetal Bovine Serum FBS (12103C, Sigma-Aldrich, USA), 0.1 mM *β*-mercaptoethanol (Sigma-Aldrich, USA), Interleukin 2 IL-2 human animal-component-free recombinant expressed in E. coli, ≥98% SDS-PAGE and ≥98% HPLC (Sigma-Aldrich, USA) suitable for cell cultures. All cell lines were consistently maintained in a humidified incubator at 37°C and 5% CO2.

### 2.4. Thymoquinone Cell Cytotoxicity on Tumor and NK Cells

Thymoquinone cell cytotoxicity was analyzed with a CyQUANT LDH Cytotoxicity Assay kit (C20301, Invitrogen, USA). MCF-7 cells were seeded at a density of 15 × 103 cells per well in a 96-well flat-bottom plate and incubated overnight. Then, NK cells were cocultured at an effector cell/target cell (E/T) ratio of 1 : 2 in the presence of different thymoquinone concentrations (25 and 50 *μ*M) for 5 h. A CytoTox 96 lysis buffer was added and incubated for 45 min in an incubator. CytoTox 96 reagent was added to each well and incubated for 30 min at room temperature in the dark, and then, a stop solution was added. Absorbance was measured at 680 and 490 nm. LDH activity was measured by subtracting the 680 nm absorbance value (background) from 490 nm absorbance [36]. Cytotoxicity % was calculated by using the formula:

(1) $$\begin{array}{c} \frac{\text{compound}\_\text{treated LDH activity}-\text{spontaneous LDH activity}}{\text{maximum LDH activity}-\text{spontaneous LDH activity}}\times 100. \end{array}$$

### 2.5. NK cell activity determination

MCF-7 and NK cells were cocultured in the presence of thymoquinone for 5 h. Cell-free supernatants were harvested to measure the production of IFN-*α* (244304-026, Invitrogen, USA) granzyme B (E0899Hu, BT lab, UK), and perforin/pore-forming proteins (E0070Hu, BT lab, UK) using a human enzyme–linked immunosorbent assay kit. Absorbance was measured at 450 nm using a microplate reader (Multiskan G0 1.00.40, Thermo, serial no. 1510-03131C) [37]. The concentrations of the unknowns were calculated from standard curves Figure S2.

### 2.6. Statistical Analyses

The data were processed using GraphPad Prism 9 and the results were presented as the mean ± SD of three independent experiments. Statistical significance was tested using one-way analysis of variance (ANOVA) and Tukey's multiple comparison test to identify significant differences between groups, with *P* < 0.05 considered significant.

## 3. Results

### 3.1. Cytotoxic Effect of Thymoquinone on NK Cells against MCF-7 Cells

**Figure 1:**
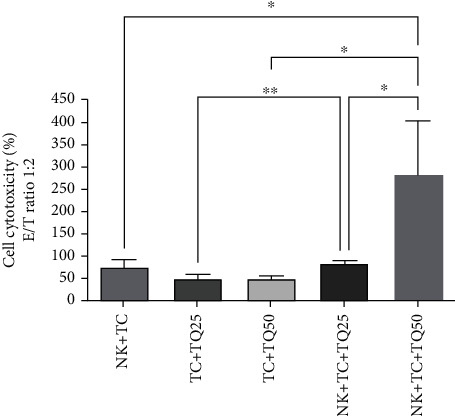
NK cell cytotoxicity against MCF-7 cells in the presence or absence of thymoquinone. Fifty *μ*M of thymoquinone upregulated NK cell cytotoxicity in MCF-7 cells significantly compared to the control cells (NK + TC) at P < 0.04 (one-way ANOVA, Tukey's test). Cell cytotoxicity significantly increased in TC + TQ25 + NK and TC + TQ50 + NK compared to TC25 and TC + TQ50 at P < 0.0064 and P < 0.0251, respectively (one-way ANOVA, Tukey's test). There was a significant increase in NK cell cytotoxicity between the two doses of thymoquinone (25 and 50 *μ*M) at P < 0.04 (one-way ANOVA, Tukey's test). All data are expressed as mean ± SD of three independent experiments.

The target NK cell cytotoxicity was evaluated in a coculture with MCF-7 cells. The effector (NK cells) and target (MCF-7 cells) ratio was 1 : 2 and different concentrations of thymoquinone (25 and 50 *μ*M) were added to the cultures. In the presence of thymoquinone, there was a significant increase in NK cell cytotoxicity. In particular, 50 *μ*M thymoquinone increased NK cell cytotoxicity in MCF-7 cells to the maximum effect of 283.244%, as compared with the control NK cells cocultured with tumor cells (NK + TC) at 77.69% (one-way ANOVA, *n* = 3, *P* < 0.04). Cell cytotoxicity was also significantly increased in tumour cells treated with both 25 *μ*M and 50 *μ*M thymoquinone in the presence of NK cells, as compared to tumor cells treated with the same thymoquinone concentrations in the absence of NK cells (one-way ANOVA, *n* = 3, *P* < 0.0064, and *P* < 0.0251, respectively). In addition, there was a significant increase in NK cell cytotoxicity between the two doses of thymoquinone (25 and 50 *μ*M; one-way ANOVA, *n* = 3, and *P* < 0.04), as seen in Figure 1.

### 3.2. Effect of Thymoquinone on NK Cell Activity

**Figure 2:**
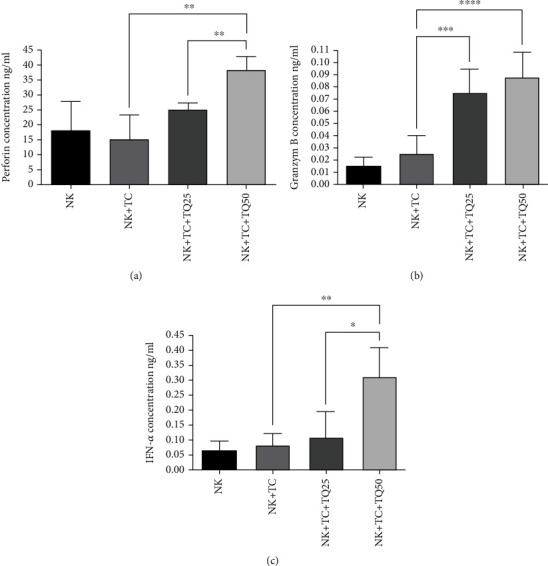
Major cytokines against MCF-7 cells, as produced by NK cells affected by thymoquinone. (a) The increased production of perforin from NK cells when cocultured with tumor MCF-7 cells treated with 50 *μ*M thymoquinone compared to both the control (NK + TC) and NK cells cocultured with tumor cells treated with 25 *μ*M (P < 0.0041 and P < 0.0019, respectively; one-way ANOVA, Tukey's test). (b) The enhanced production of granzyme B from NK cells cocultured with tumor cells treated with both thymoquinone concentrations (50 and 25 *μ*M) compared to the control (NK + TC; P = 0.0001 and P < 0.0001, respectively; one-way ANOVA, Tukey's test). (c) illustrate the increased production of interferons in NK cells cocultured with tumor cells treated with 50 *μ*M thymoquinone compared to both the control and NK cells cocultured with tumor cells treated with 25 *μ*M (P < 0.007 and P < 0.04, respectively; one-way ANOVA, Tukey's test). All data are expressed as mean ± SD of three independent experiments.

Thymoquinone enhances the release of perforin, granzyme B, and IFN-*α*, the major secreted cytokines of NK cells. In accordance with this understanding, the NK cells cocultured with MCF-7 cells and treated with 50 *μ*M thymoquinone had significantly stimulated perforin production compared with the control NK cells cocultured with tumor cells (one-way ANOVA, *n* = 3, *P* < 0.0041, Tukey's). The higher 50-*μ*M thymoquinone dose significantly enhanced the NK cells' perforin production over the lower 25-*μ*M dose (one-way ANOVA, *n* = 3, *P* < 0.0019, Tukey's), as seen in Figure 2(a). In turn, the NK cells' granzyme B production was significantly increased in the presence of 25 and 50 *μ*M thymoquinone compared with the control tumor cells in the presence of NK cells alone (one-way ANOVA, *n* = 3, *P* = 0.0001, and *P* < 0.0001, respectively, Tukeys), as seen in Figure 2(b). IFN-*α* production also significantly increased in tumor cells treated with 50 *μ*M thymoquinone compared with control tumor cells and in tumor cells treated with 25 *μ*M thymoquinone in the presence of NK cells (one-way ANOVA, *n* = 3, *P* < 0.007, and *P* < 0.04, respectively, Tukey's), as seen in Figure 2(c).

**Figure 3:**
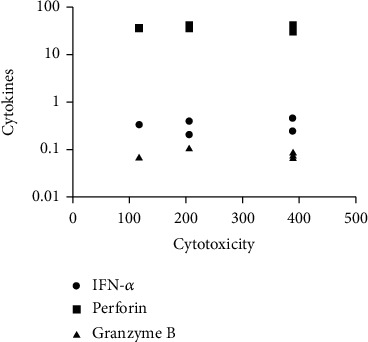
The plot of the Spearman correlation coefficient (2-tailed) between perforin, IFN-*α* and granzyme B cytokines and NK cytotoxicity. There was no significant correlation between perforin, IFN-*α* and granzyme B and NK cytotoxicity.

The correlation between individual cytokines and NK cytotoxicity was analyzed using Spearman correlation coefficient. There was no significant correlation between any of the cytokines and NK cytotoxicity although perforin production was the highest among them as seen in Figure 3.

## 4. Discussion

Many studies report the immunomodulatory effect of thymoquinone on NK cells [38–41]. The effect of thymoquinone on various immune cells is well-investigated, since inflammatory immune cells, such as NK cells, are considered part of the complex microenvironment that forms both primary and metastatic tumors [42]. Despite this, the mechanism that triggers thymoquinone's effect on cancer cells is not well-known. Moreover, it is not yet determined whether the anticancer effect of this active compound acts on the cancer cells itself or enhances NK cell anticancer activity.

In this study, we investigated the effect of two concentrations of thymoquinone on the cytotoxicity of NK cells and its anticancer activity using the MCF-7 breast cancer cell line. The results demonstrate that treating cancer cells with a high dose of thymoquinone in the presence of NK cells enhanced NK cell cytotoxicity more than NK cells cocultured with cancer cells alone or in cells with a lower dose of thymoquinone. In addition, NK cell cytotoxicity was significantly higher when used to treat cancer cells cocultured with either thymoquinone concentration compared to cancer cells treated with thymoquinone alone. This indicates that thymoquinone's antitumor effect is associated with its stimulation of NK cell function. This finding is similar to a study done by Shabsoug et al. who conclude that using an aqueous extract of *Nigella sativa* significantly enhanced the cytotoxic activity of NK cells isolated from human blood against a lymphoblast cell line (K-562) in vitro [43]. Our findings are also similar to an in vivo study done on mice with lymphoma, which were given an oral aqueous extract of *Nigella sativa* for 1 week. These results show that *Nigella sativa* caused a significant increase in splenic NK cell numbers, as associated with increased cytotoxic activity against Lymphoma YAC-1 tumor cells [44]. These findings are in an agreement with another in vitro study on the treatment of the same YAC-1 cell line with fresh aqueous *Nigella sativa* extract [45]. In addition, Majdalawieh et al. reported that treating YAC-1 cells with different concentrations of aqueous *Nigella sativa* extract significantly enhanced YAC-1 cell death upon amplifying NK cell cytotoxic activity, rather than the direct cytotoxic effect on the cancer cells themselves. This is also evidenced by our findings, suggesting that thymoquinone has no direct cytotoxic effect on MCF-7 cancer cells in the absence of NK cells [46].

The enhancement of the cytotoxic potential of NK cells against cancer cells is at least one proposed mechanism reported by other researchers using other plant extracts to examine their antitumour actions [46]. Our results show that thymoquinone stimulated the production of perforin, granzyme B and interferons in NK cells. These findings explain the enhanced cytotoxic potential of NK cells and its antitumour activity [43]. As mentioned earlier, our results are in line with Shabsoug, who report an increase in the production of interferons and granzyme B in NK cells after treating them with different concentrations of an aqueous *Nigella sativa* extract; as such, K-562 cancer cell death was significantly improved. In general, NK cells may eliminate different types of cancer cells through various cytotoxic mechanisms [43]. NK cells secrete many cytotoxic granules containing perforin and granzymes, which cause cell lysis. They also release sufficient amounts of cytokines, including IFN-*α*, thus increasing their cytotoxicity [47]. Moreover, NK cells express tumor necrosis factor–related, apoptosis-inducing ligand family TRAIL, and FAS ligand (FASL), which interact with TRAIL receptors and FAS ligands in cancer cells, respectively. This interaction results in cell death, signaling complex stimulating apoptosis [47]. The final mechanism involves the presence of CD16-activating receptors on NK cells, which recognize Fc on human IgG1 antibodies and trigger ADCC [48].

However, many tumors develop mechanisms to escape NK cell immunoresponses by modifying their cell surface molecules, which are involved in the recognition and release of the soluble factors that cause immunosuppressive reactions, such as TGF-*β*, prostaglandin E2 (PGE2), and IL-10 [49]. NK cell function can also be suppressed by tumor-associated fibroblasts (TAFs), the most abundant cell type within the stroma of many cancer types [50–52]. TAF/NK cell cross-talk results in inhibited NK cell activity through the release of PGE2, which in turn downregulates the expression of NKG2D, NKp30 and NKp44, and decreases NK cells' perforin/granzyme B production [53–55]. Therefore, the aim of much research has been to find therapeutic approaches to post/restore NK cell activity against cancer [56]. This research, together with other in vitro and in vivo studies, suggests that thymoquinone, the active component of *Nigella sativa*, may act as an immunomodulatory agent that enhances anticancer immune activity. It is worth mentioning that several investigations reveal that TQ may also act as a complementary therapy together with chemotherapy and radiation, thus enhancing treatment outcomes and minimizing their side effects [57–59]. This study confirmed the growth inhibitory effect of thymoquinone on human breast carcinoma MCF-7 cells, which is in agreement with previous studies. We also observed that thymoquinone treatment directly activates the cytolytic activity of NK cells against MCF-7 human breast cancer.

Our results provide strong evidence for the direct immune-stimulating effect of thymoquinone on NK cells. Even though some of the signaling molecules involved in mediating thymoquinone immunostimulatory effect in NK cells have been identified, the exact signaling pathways and molecular targets in them are still unknown. Future in vitro and in vivo studies are thus required to identify the target receptors and intracellular and extracellular factors that play certain roles in the signal transduction pathways that TQ modifies in NK cells.

## 5. Conclusion

In summary, our study showed that thymoquinone promotes the cytotoxic activity of NK cells against breast cancer MCF-7 cells. Furthermore, thymoquinone's activation of perforin, granzyme B, and interferon proteins appears to be responsible for the NK cells enhanced anticancer activity, which resulted in killing MCF-7 cells.

Future studies should focus on finding the connection between the anticancer effect of *Nigella sativa* extracts and preclinical and clinical tumor inhibition/therapy and the molecular mechanisms involved in NK activation. NK cell memory could assist in developing good NK cell anticancer protocol, since it could prove advantageous for treatments.

## Data Availability

No additional data are available.

## Supplementary Materials

Supplementary 1 Figure S1: NK cells were isolated using CD56+ selection and the purity of NK cells was assessed by flow cytometric analysis of cells stained with CD56-PE

Supplementary 2 Figure S2: NK dot plot before treatment and indicating the purity of NK cells to be 95%. IFN-*α*, granzyme B and perforin cytokines were measured by using a human enzyme–linked immunosorbent assay kit and the concentration of these cytokines were calculated using standard curve.
