# Supplementary figures and images for: Studying the Anticancer Effects of Thymoquinone on Breast Cancer Cells through Natural Killer Cell Activity
Source: Biomed Res Int. 2022 Sep 20;2022:9218640. doi: 10.1155/2022/9218640 (PMC9527111; doi:10.1155/2022/9218640)

Figure S1: NK dot plot before treatment


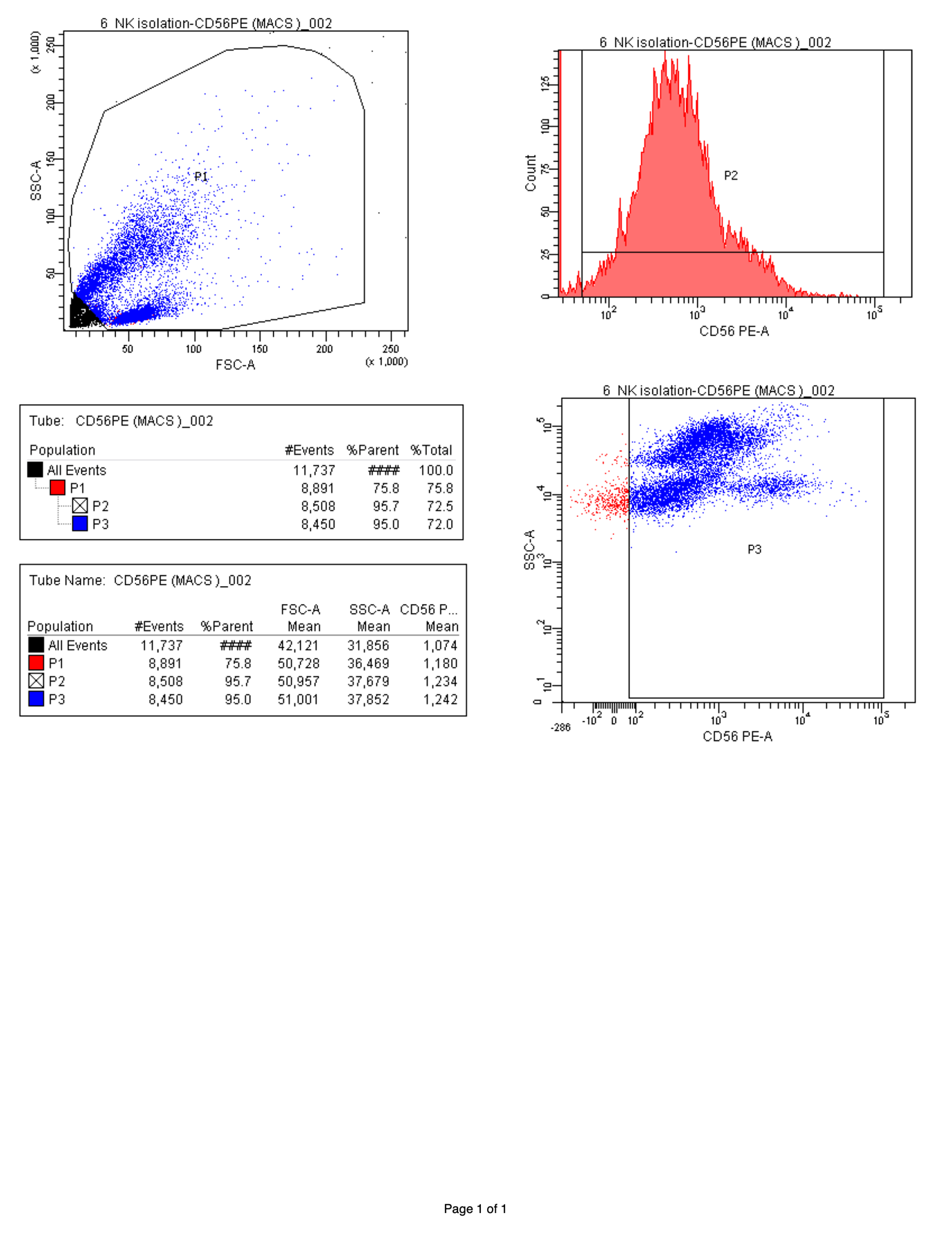

Supplement: Supplementary 1 — Figure S1: NK cells were isolated using CD56+ selection and the purity of NK cells was assessed by flow cytometric analysis of cells stained with CD56-PE [file 9218640.f1.docx]

Figure S2: cytokines standard curve


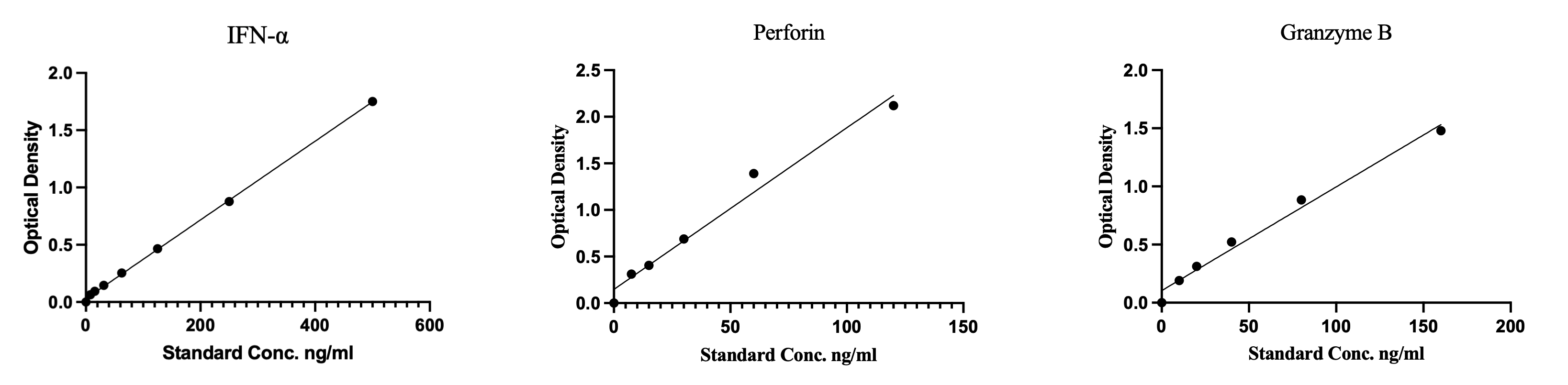

Supplement: Supplementary 2 — Figure S2: NK dot plot before treatment and indicating the purity of NK cells to be 95%. IFN-α, granzyme B and perforin cytokines were measured by using a human enzyme–linked immunosorbent assay kit and the concentration of these cytokines were calculated using standard curve. [file 9218640.f2.docx]
